# *CAPOW*: a standalone program for the calculation of optimal weighting parameters for least-squares crystallographic refinements

**DOI:** 10.1107/S1600576717016600

**Published:** 2018-02-01

**Authors:** Natalie T. Johnson, Holger Ott, Michael R. Probert

**Affiliations:** a Chemistry, School of Natural and Environmental Sciences, Newcastle University, Kings Road, Newcastle NE1 7RU, UK; b Bruker AXS GmbH, Oestliche Rheinbrueckenstrasse 49, Karlsruhe 76187, Germany

**Keywords:** charge density, multipolar refinement, weighting

## Abstract

The rigorous analysis of crystallographic models, refined through the use of least-squares minimization, is founded on the expectation that the data provided have a normal distribution of residuals. Processed single-crystal diffraction data rarely exhibit this feature without a weighting scheme being applied. These schemes are designed to reflect the precision and accuracy of the measurement of observed reflection intensities. While many programs have the ability to calculate optimal parameters for applied weighting schemes, there are still programs that do not contain this functionality, particularly when moving beyond the spherical atom model. For this purpose, *CAPOW* (calculation and plotting of optimal weights), a new program for the calculation of optimal weighting parameters for a *SHELXL* weighting scheme, is presented and an example of its application in a multipole refinement is given.

## Abstract 2

A new standalone program to calculate optimized values for weighting parameters, *CAPOW*, is presented along with enhanced visualization tools for analysing statistical distributions of data.

## 1. Introduction   

When experimentally determined crystallographic data are reduced to give reflection intensities, the degree of ambiguity in the precision of a measurement is described by its standard uncertainty. These uncertainties are calculated during the data reduction process (Schwarzenbach *et al.*, 1995 ▸) for every reflection measured. However, it is useful to consider the reliability of the calculation of the uncertainties and how they should be applied when interpreting models derived by fitting the data. The standard uncertainties for each measurement can be obtained by the summation of many, small, random errors and/or error propagation (Birge, 1939 ▸) within the data collection and processing. These standard uncertainty errors are usually calculated by the data processing software, although they can be evaluated from the analysis of multiple measurements of the same reflection (Blessing, 1987 ▸). It is expected that with a full and complete treatment of errors within the data collection and processing these will tend towards a normal distribution, in accordance with the central limit theorem. Therefore, crystallographic refinement programs have been designed to allow the application of weighting schemes, to produce a more normal distribution of residuals resultant from a refined data set. These schemes are used to calculate a value that represents the weight of each reflection in a least-squares minimization (Schwarzenbach *et al.*, 1989 ▸). The choice of weighting scheme employed is left to the user but is often dependent on the type of structural refinement being implemented. Those most generally exploited in modern crystallographic refinements can take various forms; for example, the weighting can be the same for each reflection (unit, *w* = 1), based only on the standard uncertainty of a reflection (statistical, *w* = 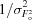 where 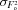 is the standard uncertainty of the reflection) or more complex, with additional contributions from the intensity and/or resolution (Spagna & Camalli, 1999 ▸).

Advanced weighting schemes can be applied to give an improved distribution of residuals if the refined structural model is understood to be complete and correct. The potential weighting schemes that can be applied, unless direct manipulation of the standard uncertainties occurs, are dependent upon what is available within the refinement packages being used. One popular weighting scheme for small-molecule crystallography is employed in the *SHELX* suite (Sheldrick, 2015 ▸). The *SHELXL* weighting scheme has six variables which can be defined by the user (*a*–*f*). For a refinement on *F* ^2^, 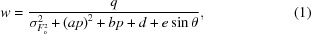
where 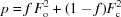 and *q* varies depending on the sign of parameter *c*:


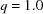
 when *c* = 0,


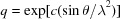
 when *c* > 0, and


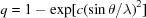
 when *c* > 0.

Here, λ is the wavelength of the X-ray radiation.

Optimal values for the *a* and *b* parameters are routinely calculated for data refined in native *SHELXL* refinements, with the other parameters remaining fixed (*c*, *d*, *e* = 0 while *f* is set to 1/3, which has been shown to reduce the bias of the weighting scheme as opposed to using *F*_c_ or *F*_o_ alone; Wilson, 1976 ▸). While many refinement packages have routines to obtain values for *a* and *b* there are some programs that do not have this functionality, especially when going beyond the spherical atom model. Therefore, in order to use this scheme effectively and increase confidence in the uncertainties on parameters derived in these situations, a method for calculating the optimal values is required. Herein we present a program, *CAPOW*, that allows the calculation of these parameters optimized to produce a minimized variance of residuals, for use in the *SHELXL* weighting scheme, and demonstrate its application in an aspherical atom refinement.

## 2. Program summary   

*CAPOW*, a program for the calculation and plotting of optimized weights, is written in Python 2.7 (Oliphant, 2007 ▸). The program can calculate the optimized *a* and *b* values for a *SHELXL* scheme using a structure factor file from a completed refinement. A graphical user interface (GUI) has been created to allow straightforward operation of the program and provide informative output. The current version of *CAPOW* features a window with two different tabs: one for the calculation of the optimal weighting scheme and another for the creation of a normal probability plot (Abrahams & Keve, 1971 ▸) of data from a crystallographic refinement.

Normal probability plots have previously been used to determine the distribution of residuals and hence assess the quality of crystallographic refinements and the data they rely upon (Zhurov *et al.*, 2008 ▸; Henn & Meindl, 2016 ▸). These plots should demonstrate a normal distribution, if the structural model is correct and errors in the measurements and applied weights have been evaluated correctly, because of the expected tendency of residuals towards a normal distribution as in the central limit theorem. Residuals from a refinement are sorted in ascending order and plotted against the residual value that would be expected if the residuals have a normal distribution. The residuals are described as being normally distributed where a gradient of 1 and an intercept of 0 are observed.

While least-squares minimization methods do not depend upon a normal distribution of residuals, the refinements are not robust to cases where these residuals are not normally distributed (Prince, 2004 ▸). Furthermore, uncertainties for any parameters derived from the model, such as atom positions, bond lengths or molecular properties like dipole interactions, are calculated with the assumption that the residuals are normally distributed. The presence of a non-normal distribution of residuals can imply the existence of systematic errors, incorrect or incomplete modelling of the data, or error propagation within a given refinement. Such potential errors should be investigated and removed before applying a weighting scheme.

### 2.1. Calculation of optimal weighting parameters   

The optimal weighting calculation is based upon a script from the computational crystallographic toolbox (*cctbx*; Grosse-Kunstleve *et al.*, 2002 ▸). The *cctbx* is open source and freely available to download and alter. To enable easier modification and adaptation, the *cctbx*-dependent functions were rewritten to remove the requirement of the *cctbx* package for the operation of the program; however, the actual process for the optimization is largely unchanged.

The optimal weighting is determined using a grid-search method. After the initial starting values for *a* and *b* are calculated, the data are arranged in order of *F*_c_ and divided into ten equal bins. The variance of the weighted goodness of fit (wGooF) of each of the ten bins is calculated for a 9 × 9 grid of incrementally increasing *a* and *b* values. The combination of *a* and *b* that gives the minimum variance within the grid is used to give a new starting point for the next grid. The step size, *i.e.* how much *a* or *b* increases between subsequent grid points, is reduced, provided that the minimum variance did not occur at the edge of the grid. The process is repeated until a stopping condition, usually based on the size of the *a* or *b* step, is fulfilled. The values of *a* and *b* that give the minimum variance from the final grid are taken as the optimal parameters to be used in the weighting scheme. The user interface has been customized to allow a choice of both stopping condition and initial starting values for *a* and *b*.

Weights calculated using *CAPOW* were compared with those calculated in *Olex2* (Dolomanov *et al.*, 2009 ▸), a spherical atom refinement program which utilizes the *cctbx* within the olex2.refine function to calculate weighting parameters. There was a high degree of consistency between the computed values.

**Figure 1:**
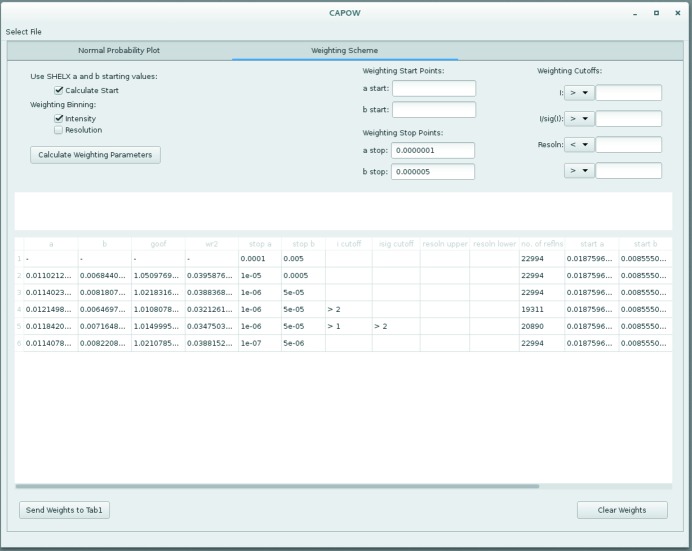
Screenshot of the ‘Weighting Scheme’ tab from the *CAPOW* GUI.

The interface to the weighting scheme calculation is displayed in Fig. 1 ▸. This interface allows the user to apply various cut-offs to the data from which the weighting scheme should be determined as well as the starting and stopping points for the grid search. Starting points can be either input manually or calculated using a *SHELXL*-style method (which is the default when the ‘Calculate Start’ check box is selected). Selected weights can then be transferred to the normal probability plot tab using the ‘Send Weights to Tab 1’ button.

### 2.2. Normal probability plots   

Normal probability plots are created using the *matplotlib* package (Hunter, 2007 ▸) and are based upon *DRKplot* (Stash, 2007 ▸). The graphs of normal probability output by *DRKplot* are calculated using the average of binned residuals within the range −4 to +4. However, to allow for the identification of potential outliers, additional features were implemented in *CAPOW*. The normal probability plots created in this program display every residual value and the option to edit the range of the axes.

**Figure 2:**
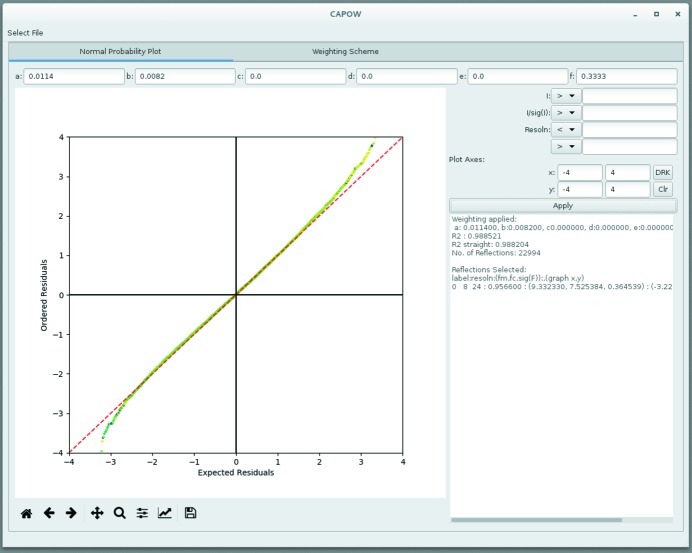
Screenshot of the ‘Normal Probability Plot’ tab from the *CAPOW* GUI.

Within the normal probability plot tab (Fig. 2 ▸), cut-offs to the data based on intensity, intensity over standard uncertainty and resolution can be applied, alongside a weighting scheme, which can be either chosen manually or calculated in the ‘Weighting Scheme’ tab. An information box allows the user to see the weighting scheme that was applied in the displayed plot. It can also display the reflection indices and structure factor information for any given data point in the plot.

### 2.3. Requirements   

The *CAPOW* GUI requires Python 2.7 and the packages *numpy*, *scipy* (van der Walt *et al.*, 2011 ▸), *matplotlib* and *pyqt* (Summerfield, 2007 ▸). Optimized *a* and *b* values are calculated for refinements on *F* ^2^, based on 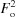, 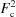 and 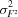 found within an .fcf (*SHELXL* LIST 4 or 8) or .fco [*XD2016* (Volkov *et al.*, 2016 ▸), a multipolar refinement program] structure factor file, containing the indices and the calculated and observed structure factors as well as the associated standard uncertainties for observed reflections. Additional information is required from a .cif file (the number of independent parameters applied to refinement) and .ins/.mas file (wavelength, weight applied and unit-cell parameters). The program outputs optimized values for both *a* and *b* to be applied in further refinement.

The source code has been tested using Scientific Linux 7.0 and Windows 8, and has been written with no operating system dependencies.

## 3. Application   

*XD2016*, and prior versions, is a widely used crystallographic refinement package for aspherical atom refinement. Atom-centred spherical harmonic (multipolar) functions are used to describe aspherical electron density distributions (Hansen & Coppens, 1978 ▸). These can reveal additional features of interest in the structural model, such as the locations of bonding and lone pair electron density.

Within the *XD2016* program, three different weighting schemes can be applied; unit, statistical or a *SHELXL* weighting scheme. Whilst a statistical weighting scheme is most often utilized when conducting charge density refinements, the success of this scheme depends upon the standard uncertainty of each reflection being calculated correctly during the data reduction and processing steps. The underestimation of standard uncertainties has been documented for multipolar refinements uncertainties (Leusser, 2012 ▸) and produces a characteristically shaped normal probability plot (Henn & Meindl, 2016 ▸). This would suggest that statistically weighting the data in the refinement of the structural model in these cases is inadequate.

There is no function to optimize the parameters for the *SHELXL* weighting scheme within *XD2016*. Weighting values calculated for a spherical atom model of the data are not transferable to a multipole refinement, owing to the differences in the predicted data from the model. Therefore, we have used *CAPOW* to calculate optimal values of *a* and *b* parameters.

**Figure 3:**
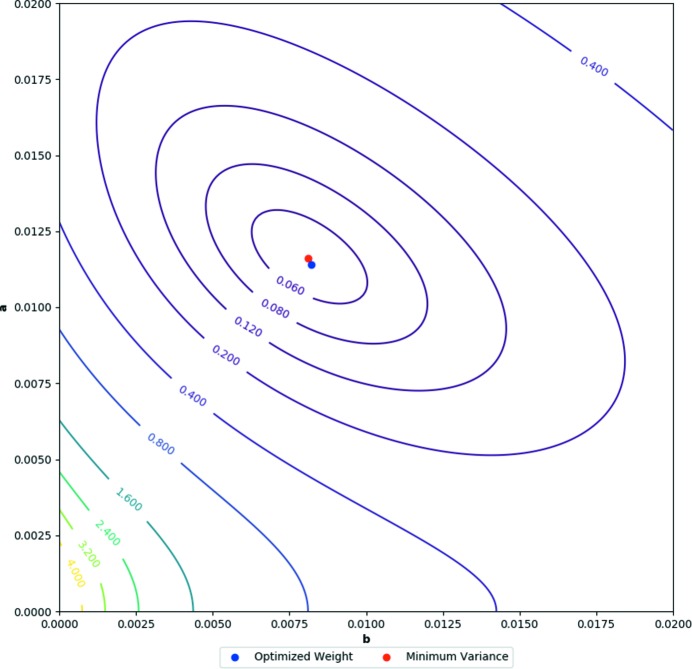
A contour plot of the variance of a *SHELXL* weighted multipole refinement calculated for a range of *a* and *b* values. The optimized weight determined by *CAPOW* and the actual minimum variance of the grid are also displayed.

To assess the applicability of a grid-search mechanism for multipolar data (as used by *CAPOW*) it was important to examine the spread of the variance of the wGooF of binned data from a charge density refinement: the value to be minimized in the weighting scheme optimization. The variance was calculated for *SHELXL* weighted data with different combinations of *a* and *b* and displayed as a contour plot (Fig. 3 ▸). The variance tends towards a single minimum between 0 and 0.02. The optimal *a* and *b* values calculated by the weighting scheme minimization correlate well to the position of the minimum of the variance within the grid.

When using data from a multipole refinement, the stopping points originally applied in the *cctbx* function (a_stop = 0.0001 and b_stop = 0.005) did not result in convergence. The calculated *a* and *b* starting values are used to determine initial grid step sizes, giving values that were smaller than the stopping conditions (thus preventing the grid search from running). To counteract this, we recommend using *a* and *b* stopping points of a_stop > 0.00001 and b_stop > 0.0005. The problem could also be overcome by changing the initial starting points for *a* and *b*. The weighting applied in this refinement was calculated with stopping points a_stop = 1 × 10^−7^ and b_stop = 1 × 10^−6^.

**Figure 4:**
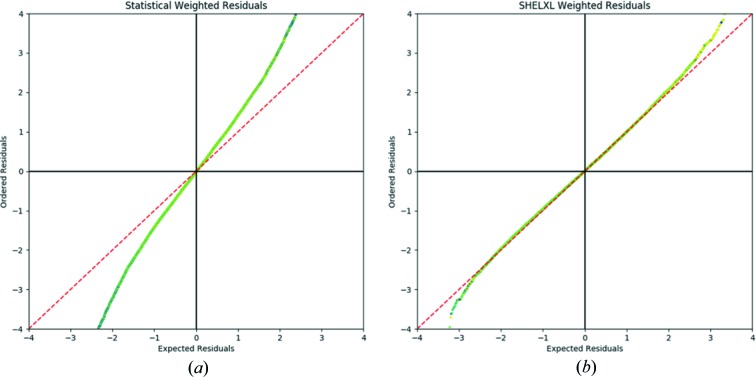
Normal probability plots generated inside *CAPOW*, showing statistically weighted residuals (*a*) and the calculated, optimized, weighting scheme (*a* = 0.0114 and *b* = 0.0082) (*b*) for a completed multipole refinement. The dashed red line indicates the expected values for normally distributed residuals.

Fig. 4 ▸(*a*) shows the normal probability plot created using statistically weighted data. The values for a normal distribution of residuals are shown as a dashed red line that can be compared with the experimental values. The deviation of the residuals from a normal distribution can be assessed by computing a line of regression using data between the lower and upper quartiles to remove bias from any large outliers. For the statistically weighted data, this gives an equation of *y* = 1.3985*x* − 0.02406. As previously stated, if the residuals were normally distributed a gradient of 1 and an intercept of 0 would be expected. The result shown, alongside visual inspection of the graph, highlights that these data do not demonstrate a normal distribution.

To apply the *SHELXL* weighting scheme, optimal parameters determined by *CAPOW* are input into the refinement master file. Several iterations of the least-squares refinement were undertaken until convergence was achieved. Optimal *a* and *b* parameters for the newly converged model are calculated and then applied. The process of calculation and refinement is repeated until convergence of the *a* and *b* parameters occurs.

The normal probability plot from the converged *SHELXL* weighted refinement (Fig. 4 ▸ *b*) is much closer to that which would be expected from a normal distribution of residuals. The line of regression of data between the lower and upper quartiles has an equation of *y* = 0.9707*x* − 0.00006, *i.e.* a gradient much closer to the ideal value of 1, with an intercept of close to zero.

Key refinement statistics from both the statistical and the *SHELXL* weighting are shown in Table 1 ▸. A reduction in the wGooF with the *SHELXL* weighting is found, giving a value much closer to 1, along with an increase in the value of the weighted residual. For the data set used in the above refinements, there was no significant statistical difference observed for atom positions, bond lengths, bond angles or multipolar populations between the statistical and *SHELXL* weighted refinements. However, the multipole parameters calculated from the *SHELXL* weighted refinement have larger standard uncertainties than the statistically weighted refinement. This causes some populations to become less statistically significant.

## 4. Conclusions   

We have demonstrated the use of a standalone weighting program, *CAPOW*, embedded within a user-friendly graphical interface, enabling the calculation of the optimal *a* and *b* values of a *SHELXL* weighting scheme. We have shown that, by applying a correctly determined weighting scheme, a more normal distribution of residuals can be produced. However, caution must always be exercised when applying a weighting scheme that is based upon a model. This situation relies on the assumption that the model is correct. Therefore, as a non-normal distribution of residuals can indicate the presence of systematic errors, it is always advisable to use statistical weights initially and eliminate as far as possible any systematic errors before applying a more complex weighting scheme.

The *SHELXL* refinement has three other parameters that could also be optimized (*c*, *d* and *e*). Further work is being conducted to analyse the impact of and to expand the optimization routines to evaluate these additional parameters.

*CAPOW* and its source code are distributed *via* the nu-xtal-tools repository which can be found at http://github.com/nu-xtal-tools.

## Figures and Tables

**Table 1:** Table of refinement parameters from statistical and *SHELXL* weighted refinements

Weighting scheme	Statistical	*SHELXL*
*R*_1_	0.0180	0.0180
*wR*_2_	0.0199	0.0289
GooF	1.6143	1.6679
wGooF	1.6143	1.0118
